# Quantum mechanical analysis of yttrium-stabilized zirconia and alumina: implications for mechanical performance of esthetic crowns

**DOI:** 10.1186/s40001-024-01851-2

**Published:** 2024-04-24

**Authors:** Ravinder S. Saini, Abdulkhaliq Ali F. Alshadidi, Vishwanath Gurumurthy, Abdulmajeed Okshah, Sunil Kumar Vaddamanu, Rayan Ibrahim H. Binduhayyim, Saurabh Chaturvedi, Shashit Shetty Bavabeedu, Artak Heboyan

**Affiliations:** 1 Department of Dental Technology, COAMS, King Khalid University, Abha, Saudi Arabia; 2 Department of Prosthetic Dentistry, College of Dentistry, King Khalid University, Abha, Saudi Arabia; 3 Department of Restorative Dental Sciences, College of Dentistry, King Khalid University, Abha, Saudi Arabia; 4 Department of Research Analytics, Saveetha Dental College and Hospitals, Saveetha Institute of Medical and Technical Sciences, Saveetha University, Chennai, 600 077 India; 5 Department of Prosthodontics, Faculty of Stomatology, Yerevan State Medical University after Mkhitar Heratsi, Str. Koryun 2, 0025 Yerevan, Armenia; 6 Department of Prosthodontics, School of Dentistry, Tehran University of Medical Sciences, North Karegar St, Tehran, Iran

**Keywords:** Mechanical properties, Optical properties, Yttrium-stabilized zirconia, Alumina, Esthetic crown

## Abstract

**Background:**

Yttrium-stabilized zirconia (YSZ) and alumina are the most commonly used dental esthetic crown materials. This study aimed to provide detailed information on the comparison between yttrium-stabilized zirconia (YSZ) and alumina, the two materials most often used for esthetic crowns in dentistry.

**Methodology:**

The ground-state energy of the materials was calculated using the Cambridge Serial Total Energy Package (CASTEP) code, which employs a first-principles method based on density functional theory (DFT). The electronic exchange–correlation energy was evaluated using the generalized gradient approximation (GGA) within the Perdew (Burke) Ernzerhof scheme.

**Results:**

Optimization of the geometries and investigation of the optical properties, dynamic stability, band structures, refractive indices, and mechanical properties of these materials contribute to a holistic understanding of these materials. Geometric optimization of YSZ provides important insights into its dynamic stability based on observations of its crystal structure and polyhedral geometry, which show stable configurations. Alumina exhibits a distinctive charge, kinetic, and potential (CKP) geometry, which contributes to its interesting structural framework and molecular-level stability. The optical properties of alumina were evaluated using pseudo-atomic computations, demonstrating its responsiveness to external stimuli. The refractive indices, reflectance, and dielectric functions indicate that the transmission of light by alumina depends on numerous factors that are essential for the optical performance of alumina as a material for esthetic crowns. The band structures of both the materials were explored, and the band gap of alumina was determined to be 5.853 eV. In addition, the band structure describes electronic transitions that influence the conductivity and optical properties of a material. The stability of alumina can be deduced from its bandgap, an essential property that determines its use as a dental material. Refractive indices are vital optical properties of esthetic crown materials. Therefore, the ability to understand their refractive-index graphs explains their transparency and color distortion through how the material responds to light..The regulated absorption characteristics exhibited by YSZ render it a highly attractive option for the development of esthetic crowns, as it guarantees minimal color distortion.

**Conclusion:**

The acceptability of materials for esthetic crowns is strongly determined by mechanical properties such as elastic stiffness constants, Young's modulus, and shear modulus. YSZ is a highly durable material for dental applications, owing to its superior mechanical strength.

**Supplementary Information:**

The online version contains supplementary material available at 10.1186/s40001-024-01851-2.

## Introduction

An esthetic dental crown is an esthetic restoration used to replace the original shape, color, size, and thickness of teeth that are damaged or weakened [1]. This dental procedure is routinely used when a tooth has extensive decay coupled with structural damage, or when the tooth lacks a cosmetically acceptable appearance [2]. The principal aim of an esthetic crown is to safeguard the damaged tooth while simultaneously improving its function and esthetics [3].

The materials used to make esthetic crowns are different, and the choice depends on the location of the tooth, chewing needs, and patient preference [4, 5]. Porcelain or ceramic crowns are natural-looking teeth that are especially suitable for the front teeth or areas of the mouth where the teeth are visible [6]. Porcelain-fused-to-metal crowns combine the esthetic appeal of porcelain with the added strength derived from a metal substructure. Zirconia crowns have become increasingly popular because of their strength and appearance. Zirconia ceramics can withstand chipping and cracking. It can be used in anterior and posterior crowns [7, 8]. For cases in which both esthetics and strength are important, the solution is porcelain-fused-to-zirconia crowns, which combine the esthetics of porcelain with the strength of zirconia and can also be used for posterior teeth [9].

The advantages of metal crowns (made from gold or metal alloys) are their strength and durability [10]. However, because of their metallic appearance, these crowns are less commonly used in visible areas of the mouth. Composite resin crowns are made of tooth-colored filling materials that can be used to create temporary crowns [11]. Although less durable compared to some materials, composite resin crowns offer an esthetically pleasing alternative [12, 13].

The choice of crown material is based on a concerted decision between the dentist and patient, considering oral health, specific tooth requirements, and personal esthetic preferences [14]. This approach allows the operator to customize the treatment for each individual patient so that the selected crown material is tailored to their own individual requirements and contributes to the functional and esthetic requirements [4]. Yttrium-stabilized zirconia (YSZ) and alumina are two types of ceramics that are frequently used to make ceramic dental crowns, with their own advantages for application in dentistry [15, 16].

The main component of YSZ is zirconium oxide (ZrO2), which is used with yttrium oxide (Y2O3) as the stabilizing agent [17]. Yttrium is added to prevent the transformation from a tetragonal to monoclinic crystal structure, thus improving its mechanical properties [18]. YSZ has excellent strength, toughness, and hardness and is a viable material for dental crowns. Its high fracture resistance protects the crown from chipping or cracking and is biocompatible with the oral environment [19]. In terms of esthetics, YSZ can be matched in color to more natural teeth, and additional translucency adds to the more natural appearance of restorations. It is suitable for both anterior and posterior teeth [20].

Alumina crowns, in contrast, are largely made of aluminum oxide (aluminum trioxide or Al_6_2O_3_), which is a ceramic that is well known for its hardness and resistance to wear [21]. Alumina exhibits notable hardness and wear resistance that contribute to its durability [22, 23]. It has excellent biocompatibility with oral tissues and can be made to match the color of natural teeth; while it is less translucent than YSZ, the esthetics of alumina crowns are continuously improved through material processing [24]. Alumina crowns are commonly used for anterior teeth where esthetics are a primary concern, and they may be chosen for cases where wear resistance is a key consideration [25, 26]. Yttrium-stabilized zirconia and alumina are suitable options for the production of esthetic dental crowns. The choice between the two materials depends on the location of the tooth, type of clinical requirement, and patient’s choice [27]. These ceramics continue to evolve as new advancements in material science become available to the dental profession, which ultimately allows dentists to provide optimized functional and esthetic outcomes in restorative dentistry [28, 29].

In this study, we comprehensively analyzed the mechanical properties, Density of states (DOS), integrated DOS, band structures, optical properties, and stress properties of yttrium-stabilized zirconia (YSZ) and alumina, specifically in the context of their application in esthetic dental crowns. The calculations were based on the computational approach of the CASTEP (Cambridge Serial Total Energy Package) code. The results were verified to provide ideas regarding the structural, electronic, and optical parameters of these materials and to identify their potential usefulness in esthetic crown applications.

## Material and methodology

The Cambridge Serial Total Energy Package (CASTEP) code [30, 31], utilizing a first-principles approach grounded in density functional theory (DFT), was employed to calculate the ground-state energy of the materials. The generalized gradient approximation (GGA) within the Perdew (Burke) Ernzerhof scheme was used to evaluate the electronic exchange–correlation energy. Vanderbilt-type norm-conserving pseudopotentials, along with a Koelling–Harmon relativistic treatment, were applied to represent the interaction between the valence electrons and ion cores. This pseudopotential selection balances the computational efficiency with the accuracy [32, 33]. The valence electron configurations considered were 1s^2^ 2s^2^ 2p^4^ for 0, 1s^2^ 2s^2^ 2p^6^ 3s^2^ 3p^1^ for Al in alumina, and 1s^2^ 2s^2^ 2p^6^ 3s^2^ 3p^6^ 3d^10^ 4s^2^ 4p^6^ 4d^1^ 5s^2^ for Y and 1s^2^ 2s^2^ 2p^6^ 3s^2^ 3p^6^ 3d^10^ 4s^2^ 4p^6^ 4d^2^ 5s^2^ for zirconia in YSZ.

Geometry optimization for yttrium-stabilized zirconia and alumina was performed using the limited-memory Broyden–Fletcher–Goldfarb–Shanno (LBFGS) minimization scheme to achieve the lowest energy structure. A plane-wave cutoff energy of 500 eV for alumina and 625 eV for YSZ was used for the expansion. Brillouin zone (BZ) integration was conducted using the Monkhorst–Pack method, employing the k-point for alumina (3 × 3 × 1) and YSZ (2 × 2 × 2). The geometry optimization employed convergence tolerances of 10^-4^ eV/atom for total energy, 10^-2^ Å for maximum lattice point displacement, 0.03 eV Å^-1^ for maximum ionic Hellmann–Feynman force, and 0.05 GPa for maximum stress tolerance. To guarantee accurate structural, elastic, and electronic band structure property estimates while preserving the computational efficiency, finite basis set modifications were used.

## Results and discussion

### Structural properties

The structural properties of alumina were determined through a geometry optimization process employing the LBFGS (limited-memory Broyden–Fletcher–Goldfarb–Shanno) minimization scheme [34]. The optimization involved an unbounded number of LBFGS updates with a preconditioned LBFGS activated using an exponential (EXP) stabilization constant of 0.1000 and a parameter A value of 3.0000. The real lattice parameters were *a* = 4.759 Å, *b* = 4.759 Å, and *c* = 12.991 Å, with corresponding cell angles of *α* = 90.000°, *β* = 90.000°, and *γ* = 120.000°. The current volume of the unit cell was calculated as 254.803051 A^3^, resulting in a density of 2.400943 AMU/A^3^ or 3.986860 g/cm^3^. The crystal system was identified as trigonal with a hexagonal geometry. The rhombohedral centers were determined to be at coordinates (0, 0, 0), (2/3, 1/3, 1/3), and (1/3, 2/3, 2/3), corresponding to crystal class – 3 m. Additionally, the LBFGS optimization results indicated a final enthalpy of − 9.29467617 × 10^3^ eV, a final frequency of 543.62876 cm^-1^, and a final bulk modulus of 220.64766 GPa. These optimization parameters, including the estimated bulk modulus and frequency, are crucial for obtaining the lowest-energy structure of alumina, providing insights into its stable geometric configuration and overall structural characteristics.

The structural properties of yttrium-stabilized zirconia (YSZ) were also investigated through geometry optimization using the LBFGS (limited-memory Broyden–Fletcher–Goldfarb–Shanno) minimization scheme. The optimization process utilized an unbounded number of LBFGS updates with an activated preconditioned LBFGS, employing an exponential (EXP) stabilization constant of 0.1000 and a parameter A value of 3.0000. The nearest-neighbor distance, cutoff distance, and parameter mu were determined automatically, whereas the variable cell method with a fixed basis quality was employed. The optimization comprised a maximum of 2 steps, with an estimated bulk modulus of 500.0 GPa and frequency of 1668 cm^-1^. The real lattice parameters for the unit cell were identified as *a *= 5.154630 Å, *b* = 5.154630 Å, and *c* = 5.154630 Å, resulting in a cubic geometry with cell angles of *α* = 90.000°, *β* = 90.000°, and *γ* = 90.000°. The current cell volume was calculated as 136.959604 A^3^, resulting in a density of 30.452467 AMU/A^3^ or 50.567510 g/cm^3^. The crystal system was characterized as cubic, the geometry was cubic, and the rhombohedral centers were specified at the coordinates (0,0,0). The crystal class was identified as 1, and the space group as *P*1 with space number 1. The LBFGS optimization results indicated a final enthalpy of 4.89620241 × 10^5^ eV, an unchanged final frequency value from the initial value, and a final bulk modulus of 117.20470 GPa. These findings offer insight into the stable geometric configuration, crystal structure, and overall structural properties of yttrium-stabilized zirconia.

**Fig. 1:**
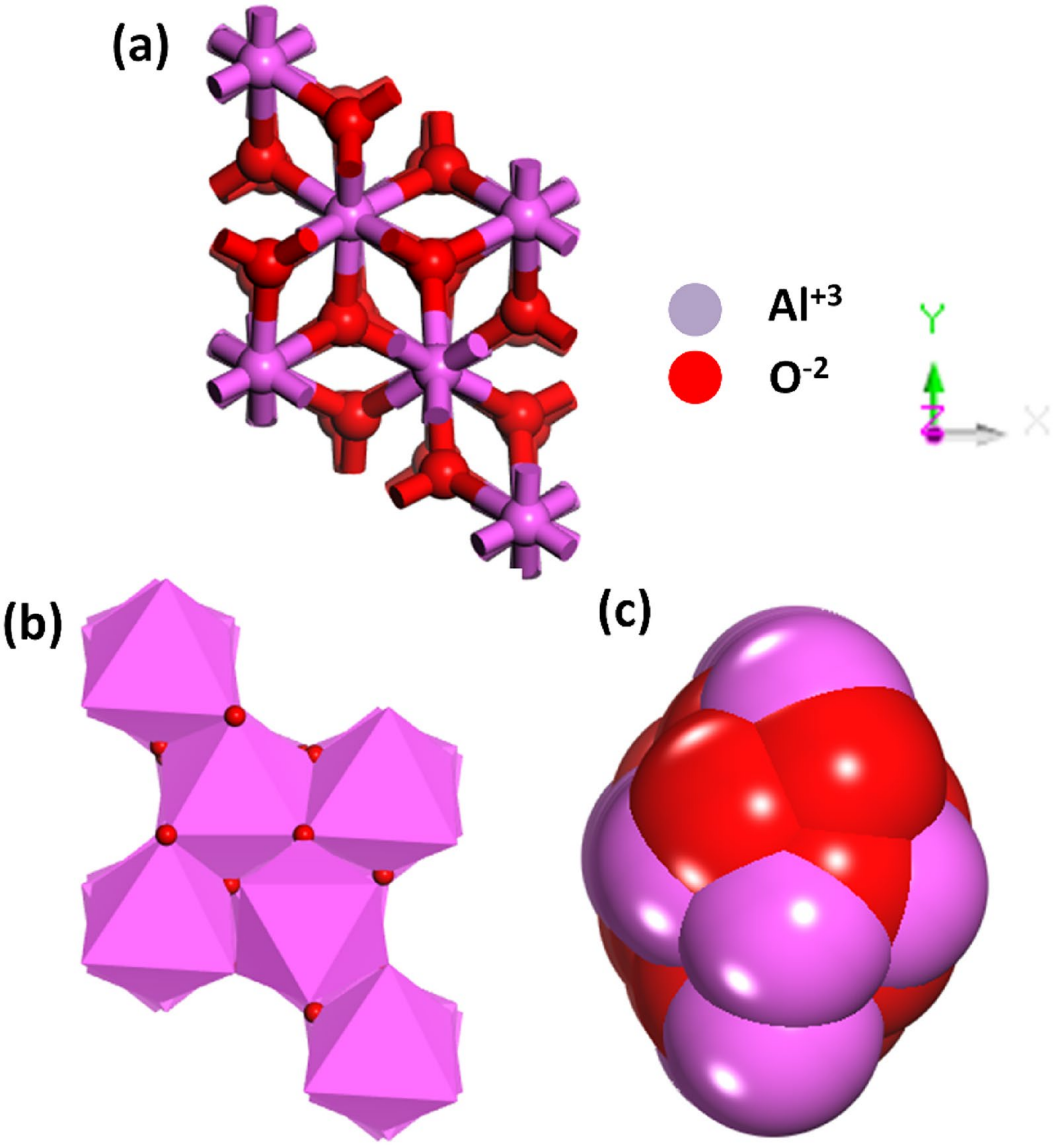
**a** Coordination environment, **b** polyhedron, and **c** charge, kinetics, and potential (CKP) of alumina

Figure 1 data offer insights into the geometry (Fig. 1(a)), polyhedron (Fig. 1(b)), and charge, kinetic, and potential (CKP) (Fig. 1(c)) energy of alumina (Al_6_2O_3_) having aluminum (Al) in the + 3 oxidation state and oxygen (O) in the − 2 oxidation state. In Fig. 1(a), the unit cell of alumina exhibits the following lattice parameters: *a* = 4.759 Å, *b* = 4.759 Å, *c* = 12.991 Å, with angles *α* = *β* = 90° and *γ* = 120°. The current cell volume was 254.803051 Å^3^ and the density was 2.400943 AMU/A^3^ or 3.986860 g/cm^3^. The crystal structure was characterized as a supercell containing three primitive cells.

In crystallography, a polyhedron is a three-dimensional geometric shape formed by connecting neighboring atoms around a central atom. As shown in Fig. 1(b), the crystal structures of alumina and aluminum atoms are typically surrounded by oxygen atoms, forming a coordination polyhedron around each aluminum center. In the polyhedron (Fig. 1(b)), alumina comprises 30 ions distributed between two species, oxygen (O) and aluminum (Al). The highest number of species was 18. The fractional coordinates of the atoms were specified by detailing their positions within a unit cell. As shown in Fig. 1(c), the potential energy density is influenced by the arrangement of the charged particles (nuclei and electrons). In alumina, the potential energy density is shaped by electrostatic interactions between the positively charged aluminum ions and negatively charged oxygen ions. The ionic character of Al–O bonds contributes to the potential energy landscape.

**Fig. 2:**
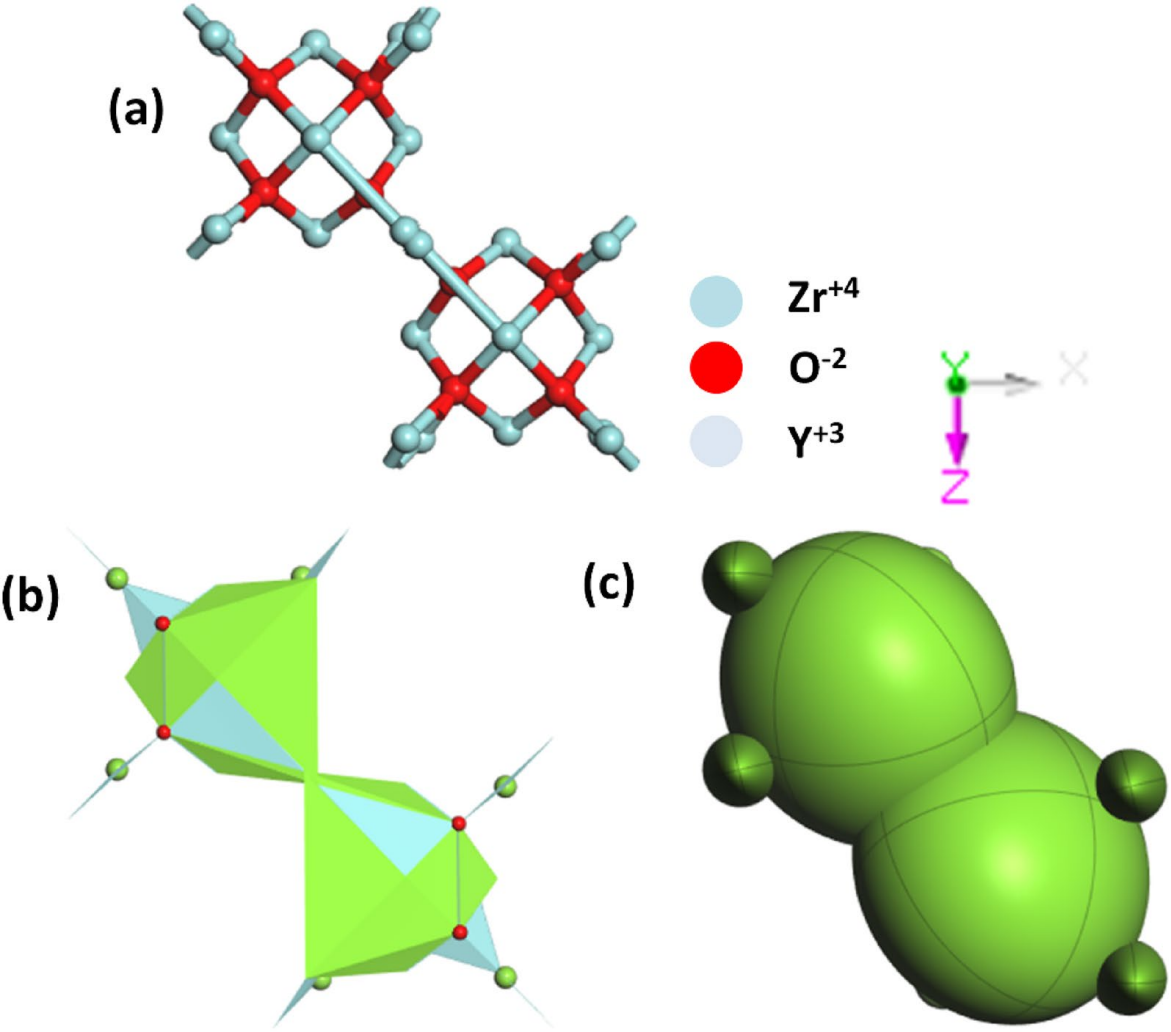
**a** coordination environment, **b** polyhedron, and **c** ellipsoid geometry of Yttrium-Stabilized Zirconia (YSZ)

The crystal structure of yttrium-stabilized zirconia (YSZ) is described in terms of its unit cell parameters (*a* = 5.154630 Å, *b* = 5.154630 Å, *c* = 5.154630 Å), as shown in Fig. 2(a). The angles between the lattice vectors were all 90°(*α* = *β* = *γ* = 90°) with the same cubic crystal system geometry. The unit cells contained oxygen (O), yttrium (Y), and zirconium (Zr). In Fig. 2(b), the polyhedron, in the context of crystallography, typically refers to a coordination polyhedron around a specific atom. In YSZ, the coordination polyhedra around Y, Zr, and O atoms depend on the crystal structure. For YSZ, the central atoms could be zirconium (Zr), yttrium (Y), or oxygen (O). The zirconium and yttrium atoms may exhibit polyhedral coordination with the surrounding oxygen atoms. Oxygen atoms typically form polyhedra around cationic species, such as Zr and Y. Ellipsoids are often associated with the electron density distribution around an atom. In the context of electronic structure calculations, an ellipsoidal representation of the charge density or electron cloud is used to describe the spatial distribution of the electrons. In Fig. 2(c), the zirconium and yttrium atoms in yttrium-stabilized zirconia (YSZ) have associated ellipsoids that describe their thermal vibrations. These ellipsoids were centered at the average positions of the Zr and Y atoms.

### Optical properties and dynamic stability of alumina and yttrium-stabilized zirconia

#### Band structure

**Fig. 3:**
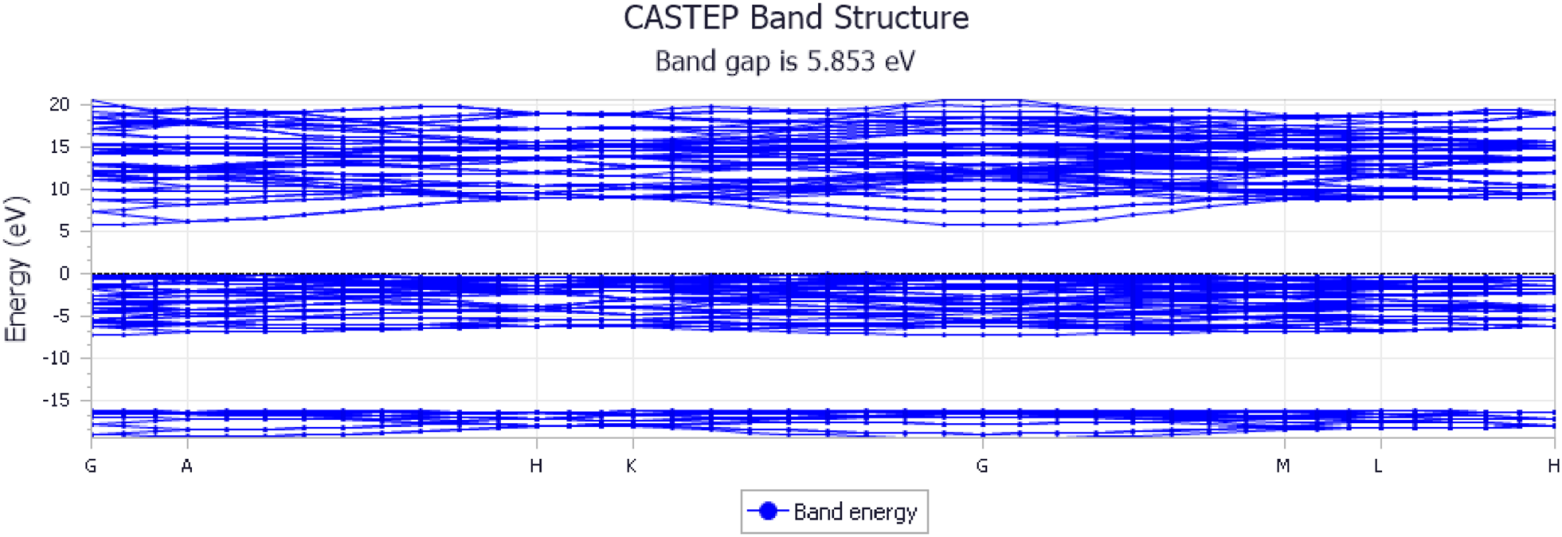
Band structure of alumina

Figure 3 shows the band structure of alumina, which provides details of its electronic properties. The X-axis of the graph shows the high-symmetry points in the Brillouin zone. In this case, they are labeled as G, A, H, K, M, and L. These points correspond to definite crystallographic directions in the reciprocal lattice of the material. The broadening at the G and A points on the axis shown in Fig. 3 suggests the fanning out (dispersion) of the electronic states at the high-symmetry points. Therefore, this can indicate electronic transitions or electronic interactions occurring at G and A. The Y-axis gives the energy in electron volt (eV) units, which encompasses—− 20 to 20 eV, the energy level associated with the electronic states of the material.

The band gap of alumina was 5.853 eV. The bandgap represents the energy difference between the top of the valence band and bottom of the conduction band. The band structure of alumina is crucial for understanding its electronic behavior. The wide bandgap of 5.853 eV [35] indicates that alumina is an insulating material, implying that it does not conduct electricity well. This property is desirable for applications such as esthetic crowns in dentistry. The insulating nature of alumina ensures that it does not interfere with electrical signals in the surrounding biological environment, making it suitable for use in dental crowns where electrical conductivity could be problematic. Overall, the band structure of alumina, with a significant band gap and specific broadening at high-symmetry points, supports its feasibility as a material for esthetic crowns, ensuring both electrical insulation and potentially favorable optical characteristics.

Moreover, the bandgap of alumina is a key factor in determining its stability. In general, materials with larger bandgaps are more stable. The bandgap represents the energy required to transition electrons from the valence to the conduction band. Alumina, which has a bandgap of 5.853 eV, is considered to have a relatively wide bandgap. A wide band gap indicates a large energy difference between the filled valence band and the empty conduction band. This large energy separation suggests that alumina is less prone to electron excitation and conductivity, making it an insulator. As an insulator, alumina is less likely to undergo spontaneous electron transitions, which contribute to its overall stability.

**Fig. 4:**
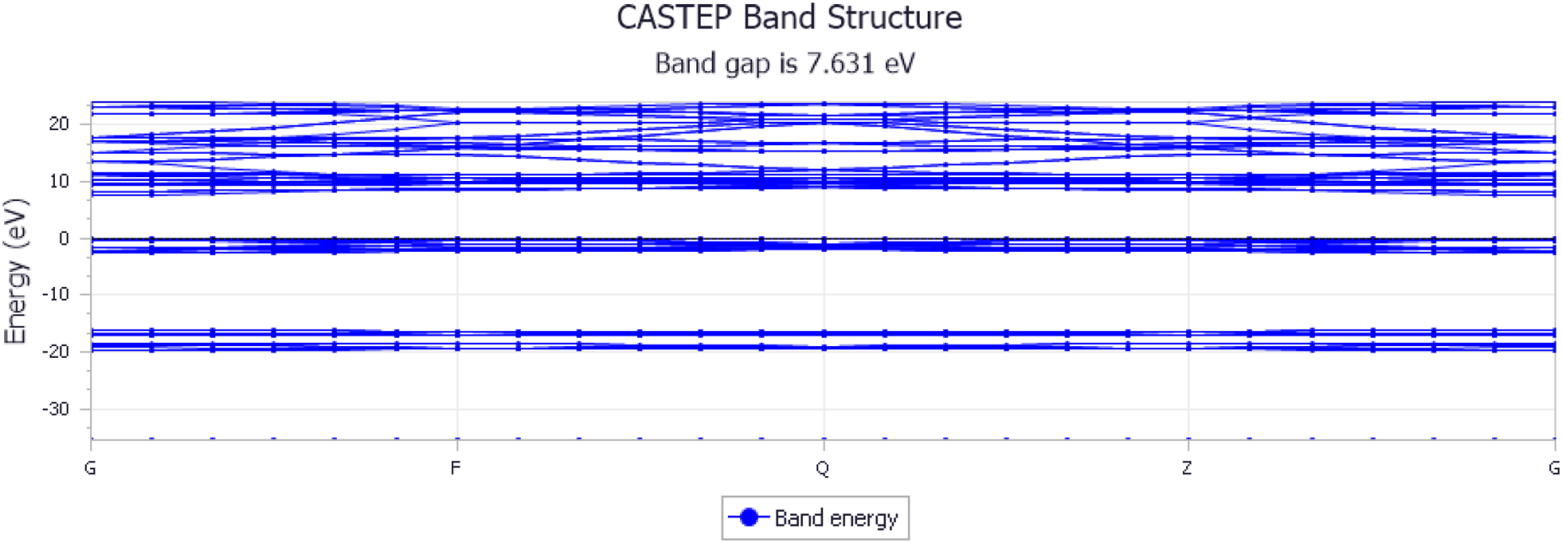
Band structure graph of Yttrium-Stabilized Zirconia

In contrast, in Fig. 4, the Y-axis represents the energy values of the electronic bands in electron volts (eV). The range was—-15 to 15 eV. In any case, the x-axis is almost the same as the previous one. The bandgap of yttrium-stabilized zirconia was 7.631 eV [36]. The bandgap represents the energy difference between the valence and conduction bands. A larger bandgap indicates a better insulation. Moreover, materials with larger bandgaps are more stable. Yttrium-stabilized zirconia (YSZ) is known for its high strength and resistance to fracture, making it a popular choice for dental ceramics, especially for esthetic crowns.

#### Refractive index

The refractive index (*n*) of a material is a dimensionless quantity that provides a quantitative description of the bending or refraction of light as it enters a material from a different medium. The refractive index is often represented as (n + i k), where (*n*) is the real part and (*k*) is the imaginary part. The real part of the refractive index (n) describes how much the speed of light in the material is lowered with respect to the speed of light in vacuum. The positive values of (*n*) imply that the material is one in which the speed of light is attenuated. The imaginary part of the refractive index (*k*) is part of the optical index, which is directly related to the absorption or attenuation of light in the material.

**Fig. 5:**
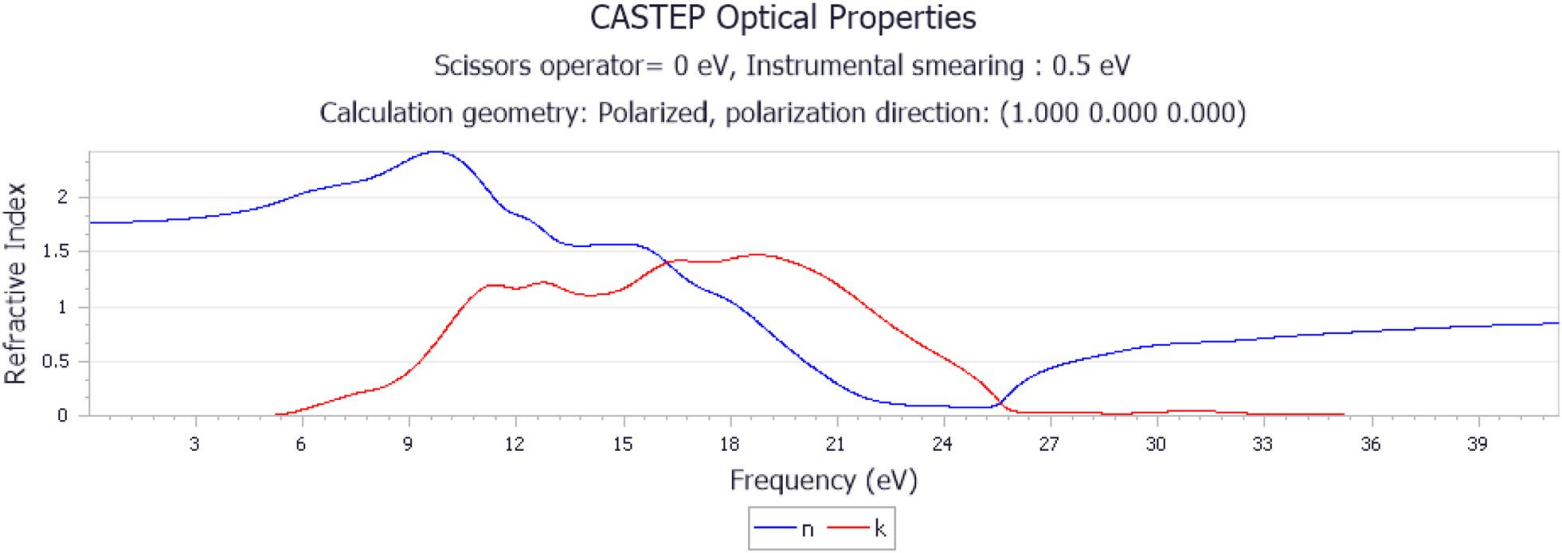
Refractive index of alumina

The real part of the refractive index, the upward trend at 9 eV in Fig. 5, suggests an increase in the refractive index, indicating increased slowing of light at this point. The downward trend at 24 Hz indicated a decrease in the refractive index, suggesting a reduction in the slowing of light. The imaginary part of the refractive index, the broadening from to the 6–24 frequency in the (*k*) values, indicates increased absorption or attenuation of light in this frequency range.

The refractive index is an important parameter in optical materials used for esthetic crown applications [37]. The positive values of (*n*) suggest that alumina can influence the speed of light, which is relevant for optical applications. The absorption indicated by (*k*) values may need to be considered, especially in esthetic applications where light transmission and appearance are crucial. Controlling the absorption properties is vital in esthetic crowns to prevent unwanted color distortions and to ensure that the crown appears natural.

The refractive index is directly associated with the dispersion of light. Achieving a harmonious color match with natural teeth requires careful control of the refractive index, particularly in the context of the broadening observed in the given frequency range. Figure 5 presents an overview of the optical behavior of alumina. The consistency of the refractive index and its response to light is critical for ensuring the optical clarity and esthetically pleasing appearance of an esthetic crown.

**Fig. 6:**
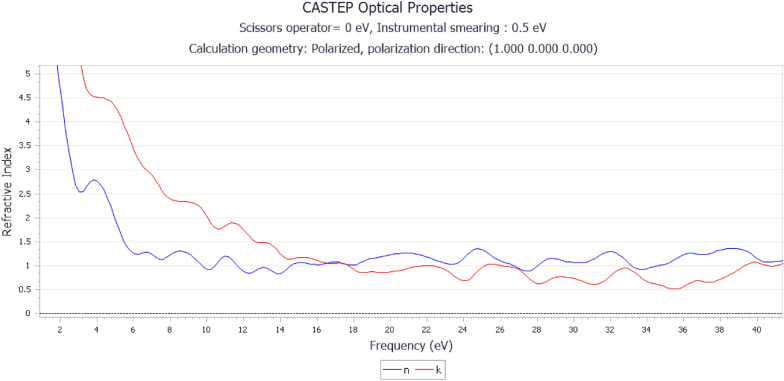
Refractive index graph of Yttrium-Stabilized Zirconia

On the other hand, the refractive-index graph in Fig. 6 for yttrium-stabilized zirconia (YSZ) provides essential information about its optical properties, shedding light on its suitability for esthetic crown. The constant value of the refractive index (*n*) in the range of 6–18 frequency indicates that YSZ maintains a consistent optical behavior within this frequency range. This consistency is beneficial for achieving uniform optical properties in esthetic crowns. The sharp decrease from 5 to 1 on the y-axis suggests a substantial change in the refractive index, which may have implications for light transmission and color perception. The subsequent upward trend to 1.5 indicates a recovery in the refractive index. The sharp downward trend in the imaginary part of the refractive index (*k*) up to 10 eV indicates low light absorption within this frequency range. This is advantageous for esthetic crowns, as it suggests minimal color distortion due to absorption. The subsequent stabilization and slight upward trend of (*k*) beyond 10 eV indicate controlled absorption properties, contributing to the stability and color accuracy of the material. A constant refractive index within certain frequency ranges is desirable for achieving optical clarity and maintaining a natural appearance in esthetic crowns. The controlled absorption properties indicated by (*k*) contribute to the prevention of unwanted color distortions, ensuring that the crown closely matches natural teeth. The consistent refractive index values and controlled absorption properties suggest the stability of the optical performance of the YSZ. This is crucial for long-term durability and esthetic success of crown restorations.

In comparison, alumina exhibits varying refractive index trends with absorption (*k*) in the observed frequency range. YSZ maintains a constant refractive index, indicating consistent optical behavior. YSZ exhibits better-controlled absorption, suggesting improved stability and color accuracy. The optical characteristics of YSZ make it a promising material for esthetic crown applications. In conclusion, yttrium-stabilized zirconia exhibits more desirable optical characteristics than alumina, making it a potentially superior material for esthetic crown applications, owing to its stable refractive index and controlled absorption properties.

### Mechanical properties

#### Stiffness matrix of alumina and yttrium-stabilized zirconia

The elastic stiffness constants (Cij) [38] of alumina, represented in GPa, provide crucial information regarding the response of the material to the applied stress and deformation, as shown in Table 1. The data in Table 1 for the elastic stiffness constants of alumina are presented for a 6 × 6 matrix.

**Table 1:** Stiffness matrix (coefficients in GPa) of alumina

417.6469	119.98878	75.42255	0.21215	21.17205	1.4208
119.98878	416.98215	72.69125	− 0.18043	− 21.1691	1.4079
75.42255	72.69125	420.14175	0.23857	0.3554	1.36645
0.21215	− 0.18043	0.23857	116.9212	0.61045	− 21.2966
21.17205	− 21.1691	0.3554	0.61045	113.7423	0.0077
1.4208	1.4079	1.36645	− 21.2966	0.0077	143.9595

The high elastic stiffness constants, particularly those of the diagonal elements (C11, C22, C33, C44, C55, and C66), suggest that alumina is mechanically stable and can withstand stress and deformation. Stability is a crucial factor in dental restorations because it ensures that the crown material can endure forces exerted during mastication without undergoing significant deformation. The off-diagonal terms (C12, C13, C23, C14, C15, C16, C24, C25, and C26) indicate the anisotropic nature of alumina. Anisotropy implies that the mechanical properties of a material vary with the direction. Anisotropic behavior is important in esthetic crowns, as it allows for tailored mechanical properties depending on the orientation of the crown and its interaction with surrounding teeth.

The elastic stiffness constants allow the material to resist wear and deformation, thereby enhancing the long-term durability of the dental restorations. The values in the matrix that contribute to the mechanical integrity of the alumina crown would permit its use for esthetic crowns that need to withstand a variety of mechanical stresses, and knowing the elastic stiffness constants becomes important when we consider the proper design and fabrication of an esthetic crown because these values will need to be able to predict how the material under study will deform to the ideal loading conditions in such a way that its performance will be optimized; on the other hand, the elastic stiffness constants (Cij) of yttrium-stabilized zirconia (YSZ), also represented in GPa, are given in a 6 × 6 matrix in Table 2.

**Table 2:** Stiffness matrix (coefficients in GPa) of yttrium-stabilized zirconia

769.58275	156.11755	156.11755	0	0	0
156.11755	769.58275	156.11755	0	0	0
156.11755	156.11755	769.58275	0	0	0
0	0	0	436.16925	0	0
0	0	0	0	436.16925	0
0	0	0	0	0	436.16925

The high values of the elastic stiffness constants, particularly those of the diagonal elements (C11, C22, C33, C44, C55, and C66), indicate that YSZ is mechanically stiff and exhibits excellent resistance to deformation under stress. High stiffness is advantageous in dental restorations because it contributes to the ability of the material to withstand forces exerted during biting and chewing. The diagonal terms of the matrix are identical, indicating isotropic behavior. Isotropy implies that the mechanical properties of the material are consistent in all the directions. Isotropic behavior simplifies the design and fabrication process for esthetic crowns, as the material responds uniformly to applied stress, ensuring predictable and reliable performance. The elastic stiffness constants influence the durability and resistance of the material to wear. YSZ’s stiffness of YSZ contributes to its ability to maintain its structural integrity over time, ensuring its long-term success as a dental restoration material. Understanding the elastic stiffness constants is crucial for designing esthetic crowns with precise mechanical properties. This enables dental professionals to tailor the material response to specific loading conditions and optimize the crown performance. While elastic stiffness is critical for mechanical performance, other factors, such as biocompatibility and esthetics, also play a role in the feasibility of YSZ for esthetic crowns. YSZ is known for its biocompatibility, and its natural color can contribute to visually appealing esthetic outcomes.

Comparing these values, YSZ has relatively higher values in its matrix than alumina, which means that YSZ is stiffer. In terms of the isotropic properties, the diagonal terms are the same for both values, indicating that they are isotropic. YSZ, which is stiffer, is more likely to have higher durability and resistance to deformation than alumina. Both materials offer precision in crown design owing to their isotropic behavior. The choice between them may depend on specific design requirements. This comparison indicates that YSZ generally has higher stiffness values, which may be advantageous in certain applications.

### Average properties of alumina and yttrium-stabilized zirconia

The feasibility of alumina for esthetic crown applications is supported by its mechanical and optical properties derived from the average properties obtained through the Voigt, Reuss, and Hill averaging schemes listed in Table 3.

**Table 3:** Average properties of alumina

Averaging scheme	Bulk modulus	Young’s modulus	Shear modulus	Poisson’s ratio
Voigt	*K*_V_ = 199 GPa	*E*_V_ = 341.6 GPa	*G*_V_ = 140.7 GPa	*ν*_V_ = 0.2139
Reuss	*K*_R_ = 198.6 GPa	*E*_R_ = 327.35 GPa	*G*_R_ = 133.58 GPa	*ν*_R_ = 0.22528
Hill	*K*_H_ = 198.8 GPa	*E*_H_ = 334.51 GPa	*G*_H_ = 137.14 GPa	*ν*_H_ = 0.21956

The mechanical strength of a material is often characterized by parameters such as Young’s modulus (*E*), bulk modulus (*K*), and shear modulus (*G*). The Young’s modulus measures a material's stiffness, indicating how much it will deform under a given load. High values of the Young’s modulus imply that the material is stiff and resistant to deformation. A high Young’s modulus indicates that alumina can maintain its shape and resist bending or flexing, which is crucial for dental crowns subjected to biting and chewing forces. The bulk modulus is a measure of the resistance of a material to volume change under pressure. The high bulk modulus values indicate that the material was resistant to compression. In dental crowns that experience pressure from biting forces, a high bulk modulus is essential for maintaining the structural integrity of the crown and preventing undesirable changes in volume. The shear modulus measures the resistance of a material to deformation caused by shear stress. This represents the ability of a material to withstand the forces that act parallel to its surface. High shear modulus values imply that the material can resist shear forces, making it mechanically robust. In dental applications, resistance to shear force is crucial for the longevity and stability of crowns during mastication. The combination of the high Young's modulus and shear modulus values indicates that alumina can provide precise and stable crown fabrication. This is important for achieving an accurate fit and long-term durability of the dental crowns.

On the other hand, the average properties of yttrium-stabilized zirconia (YSZ) provide insights into its mechanical behavior in Table 4, and these properties play a significant role in determining its feasibility as a material for esthetic crowns.

**Table 4:** Average properties of YSZ

Averaging scheme	Bulk modulus	Young's modulus	Shear modulus	Poisson’s ratio
Voigt	*K*_V_ = 360.61 GPa	*E*_V_ = 850.86 GPa	*G*_V_ = 384.39 GPa	*ν*_V_ = 0.10675
Reuss	*K*_R_ = 360.61 GPa	*E*_R_ = 832.4 GPa	*G*_R_ = 373.18 GPa	*ν*_R_ = 0.11528
Hill	*K*_H_ = 360.61 GPa	*E*_H_ = 841.66 GPa	*G*_H_ = 378.79 GPa	*ν*_H_ = 0.111

This averaging scheme provides an upper bound for the material properties. The high values of the bulk modulus (KV = 360.61 GPa), Young’s modulus (EV = 850.86 GPa), and shear modulus (GV = 384.39 GPa) indicate that YSZ is a stiff material with excellent resistance to deformation. This is advantageous for dental crowns because it suggests that YSZ can withstand forces associated with biting and chewing. This scheme provides a lower bound for the material properties. The values of the bulk modulus (KR = 360.61 GPa), Young's modulus (ER = 832.4 GPa), and shear modulus (GR = 373.18 GPa) obtained through Reuss averaging confirmed the stiffness and mechanical robustness of YSZ. The values of bulk modulus (KH = 360.61 GPa), Young’s modulus (EH = 841.66 GPa), and shear modulus (GH = 378.79 GPa) suggest that YSZ maintains a consistently high level of stiffness across the different averaging schemes.

The values of Poisson’s ratio obtained through different averaging schemes (νV = 0.10675, νR = 0.11528, νH = 0.111) suggest that YSZ has a relatively low Poisson’s ratio. A lower Poisson’s ratio is favorable for dental crowns because it indicates a lower susceptibility to deformation and better ability to maintain shape under stress. In general, the high stiffness, resistance to deformation, and low Poisson's ratio of YSZ, as indicated by its averaged properties, make it a feasible material for esthetic crowns.

In comparison, yttrium-stabilized zirconia (YSZ) exhibits a higher bulk modulus, Young's modulus, and shear modulus, along with a lower Poisson's ratio than alumina. These mechanical properties collectively suggest that YSZ is a stiffer and more resistant material, making it potentially more suitable for applications such as esthetic crowns, where mechanical strength and durability are crucial.

The eigenvalues of the stiffness matrix represent the natural frequencies at which a material vibrates when it is subjected to mechanical stimuli. In the context of alumina in Table 5, the eigenvalues of its stiffness matrix (represented by λ1 to λ6) correspond to different modes of vibration and provide insights into its mechanical behavior.

**Table 5:** Eigenvalues of stiffness matrix of alumina

λ_1_	λ_2_	λ_3_	λ_4_	λ_5_	λ_6_
105.15 GPa	109.04 GPa	155.66 GPa	302.04 GPa	358.76 GPa	598.74 GPa

The eigenvalues represent the stiffness or rigidity of alumina in different directions. Higher eigenvalues suggest higher stiffness in these specific directions, contributing to the overall stability of the material. The eigenvalues are associated with the natural frequencies of vibrations. Understanding these frequencies is crucial in applications where the material may be subjected to mechanical vibrations, ensuring that the material does not resonate or deform undesirably under specific loads.

In the context of esthetic crowns, the eigenvalues provide insights into how alumina responds to forces and stresses. Higher eigenvalues indicate a greater resistance to deformation, which is essential for maintaining the structural integrity of dental restorations.

On the other hand, in the context of yttrium-stabilized zirconia (YSZ), the eigenvalues (λ1 to λ6) provide insights into its mechanical behavior and structural characteristics in Table 6. Equal values of the first three eigenvalues (λ1, λ2, and λ3) indicate isotropic or uniform stiffness in those directions. This property is beneficial in dental applications where consistent material behavior is desired. The last three eigenvalues (λ4, λ5, and λ6) were higher, indicating an increased stiffness in specific directions. This anisotropic stiffness provides YSZ with tailored mechanical properties, making it suitable for applications in which strength and resistance to deformation are crucial.

**Table 6:** Eigenvalues of stiffness matrix of alumina

λ_1_	λ_2_	λ_3_	λ_4_	λ_5_	λ_6_
436.17 GPa	436.17 GPa	436.17 GPa	613.47 GPa	613.47 GPa	1081.8 GPa

Moreover, the higher eigenvalues in certain directions suggest that YSZ can effectively resist deformations and stresses. This durability is essential for esthetic crowns to ensure long-term performance without mechanical failure.

In contrast, the eigenvalues of the stiffness matrix highlight the mechanical differences between yttrium-stabilized zirconia and alumina. YSZ exhibits a more isotropic stiffness profile with a higher overall stiffness, making it suitable for applications that require enhanced mechanical properties, such as esthetic crowns in dentistry.

### Elastic moduli of yttrium-stabilized zirconia and alumina

**Fig. 7:**
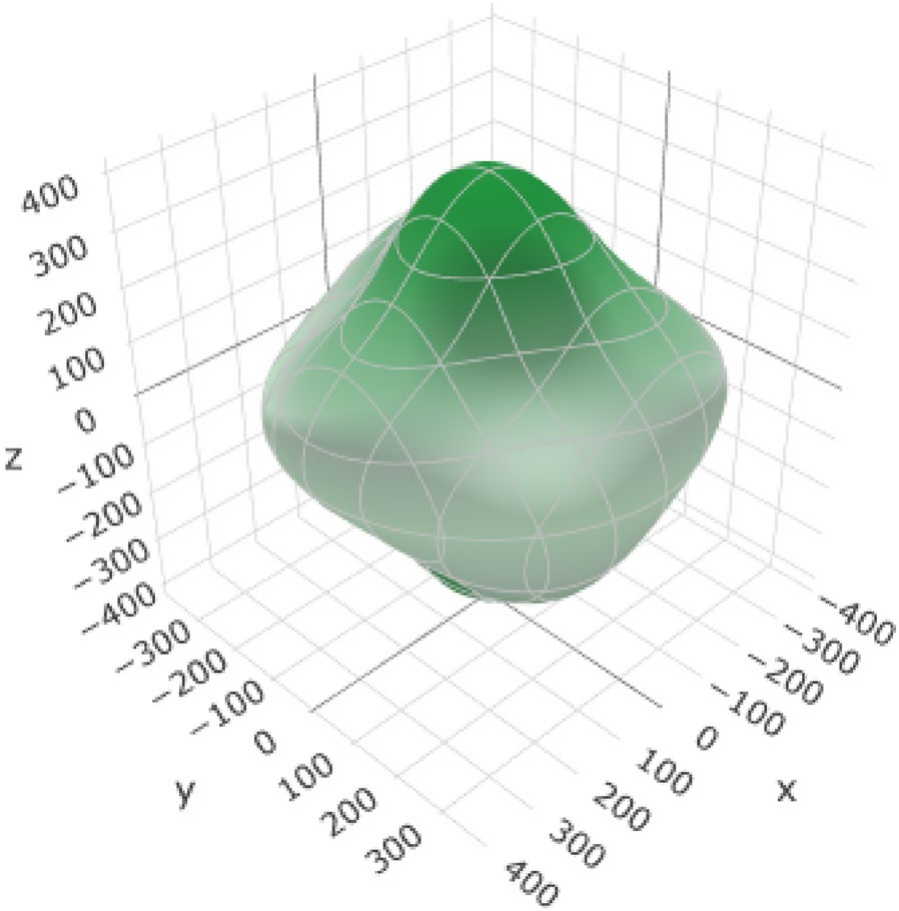
3D representation of Young’s modulus of alumina

Table 7 provides information about the variations in the elastic moduli of alumina, including the Young's modulus (Fig. 7 and (Additional file 1: Figure S1 for 2D representation), linear compressibility, shear modulus, and Poisson's ratio. These variations are crucial for understanding the response of the material to mechanical stress and play a significant role in the suitability of alumina for esthetic crown applications.

**Table 7:** Variations in elastic moduli of alumina

	Young's modulus		Linear compressibility		Shear modulus		Poisson's ratio		
	*E*_min_	*E*_max_	*β*_min_	*β*_max_	*G*_min_	*G*_max_	*ν*_min_	*ν*_max_	
Value	266.16 GPa	399.71 GPa	1.5841 TPa^–1^	1.8125 TPa^–1^	103.75 GPa	173.38 GPa	0.053073	0.3787	Value
Anisotropy	1.502		1.1442		1.671		7.1354		Anisotropy
Axis	− 0.423	0.0012	0.8251	− 0.003	0.2098	− 0.002	0.5043	− 0.633	Axis
	0.6688	0.0027	0.5647	− 0.034	0.3816	0.6798	-0.0007	− 0.009	
	− 0.617	1	0.02	0.9994	− 0.9002	− 0.733	0.8635	− 0.773	
					− 0.879	− 0.219	-0.0014	0.7763	Second axis
					0.4767	− 0.715	1	0.0016	
					− 0.0027	− 0.669	0.0017	− 0.633

The range from *E*_min_ (266.16 GPa) to *E*_max_ (399.71 GPa) represents the variation in Young's modulus. This variation describes the stiffness of the material and its ability to withstand deformation under an applied stress. An anisotropy value of 1.502 indicates that the stiffness of the material varied in different crystallographic directions.

**Fig. 8:**
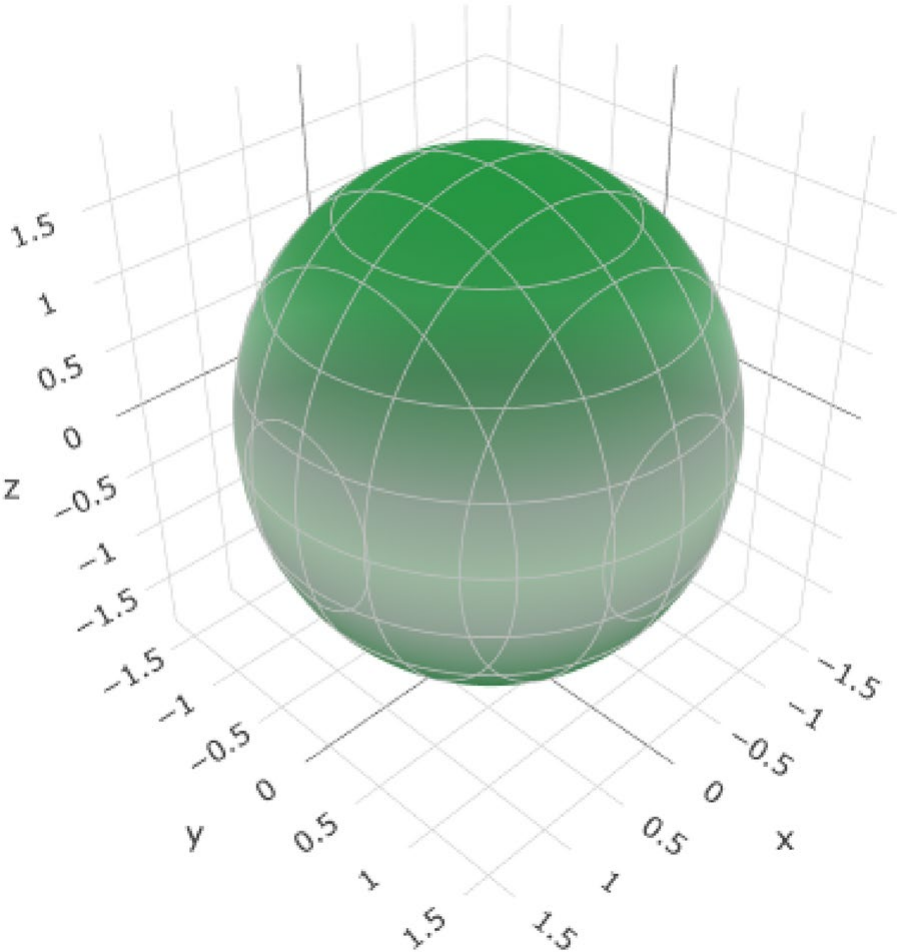
3D representation of linear alumina compressibility

**Fig. 9:**
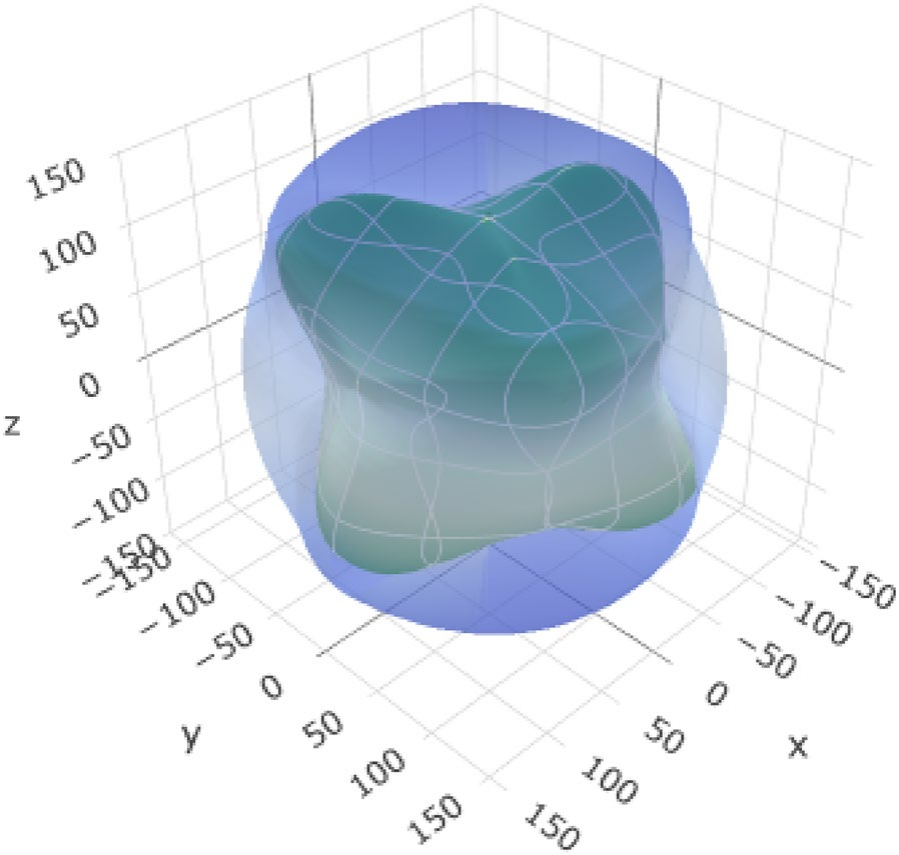
3D representation of the shear modulus of alumina

**Fig. 10:**
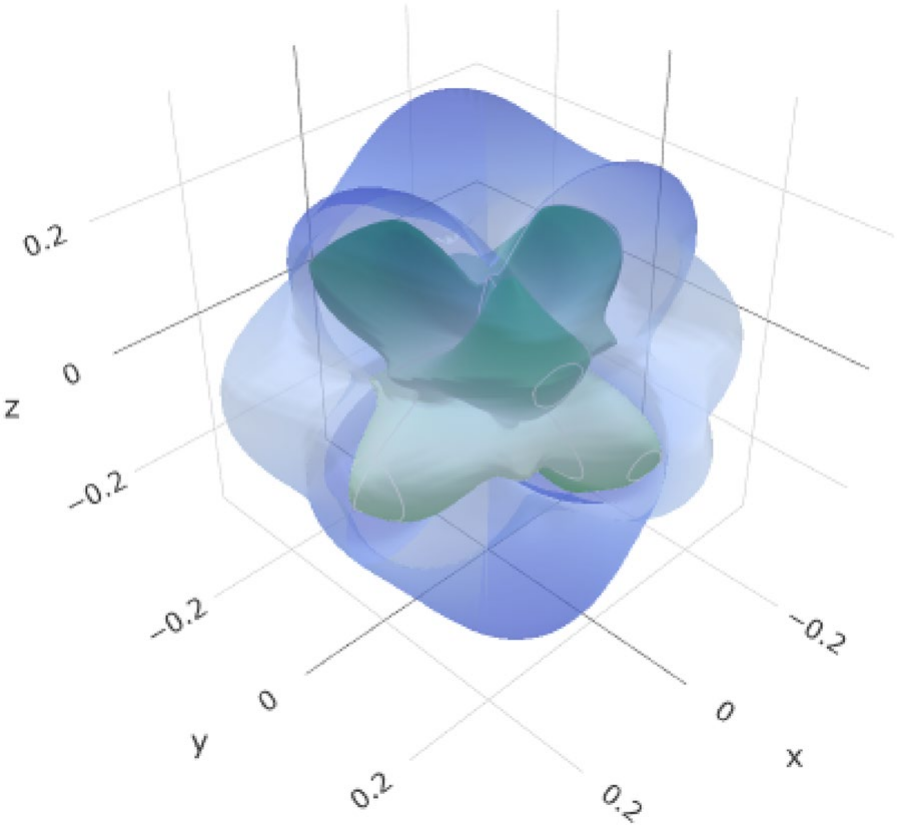
3D representation of Poisson’s ratio of alumina

The variation from *β*_min_ (1.5841 TPa^–1) to *β*_max_ (1.8125 TPa^–1) represents the linear compressibility of alumina (Fig. 8 and (Additional file 1: Figure S2 for 2D representation). This property indicates that the material responds to compressive stress. Furthermore, the range from G_min_ (103.75 GPa) to *G*_max_ (173.38 GPa) represents the variation in the shear modulus (Fig. 9 and (Additional file 1: Figure S3 for 2D representation). The shear modulus reflects the resistance of a material to deformation under shear stress. Moreover, the range from *ν*_min_ (0.053073) to ν_max_ (0.3787) represents the variation in Poisson’s ratio (Fig. 10 and (Additional file 1: Figure S4 for 2D representation). This ratio describes the tendency of the material to contract laterally when longitudinally compressed.

The anisotropy values for each property indicate the extent to which the material varies in different crystallographic directions, and the axis values indicate the orientation of the crystallographic axes with respect to the measurement axes. Nonetheless, this anisotropy allows the stiffness of alumina to be tailored in different directions. For dental applications, crown materials must closely resemble the mechanical properties of the natural teeth. The anisotropy values and axis information are helpful during fabrication to orient the crown with respect to the optimized mechanical properties of the material for the given directions.

In conclusion, variations in the elastic moduli of alumina are vital for tailoring the mechanical properties of materials to meet the specific requirements of esthetic crown applications. These properties ensure that the crown exhibits appropriate stiffness, deformation response, and dimensional stability, thereby contributing to the overall success of the dental restorations.

However, variations in the elastic moduli of yttrium-stabilized zirconia (YSZ) are important for esthetic crown applications for several reasons.

**Fig. 11:**
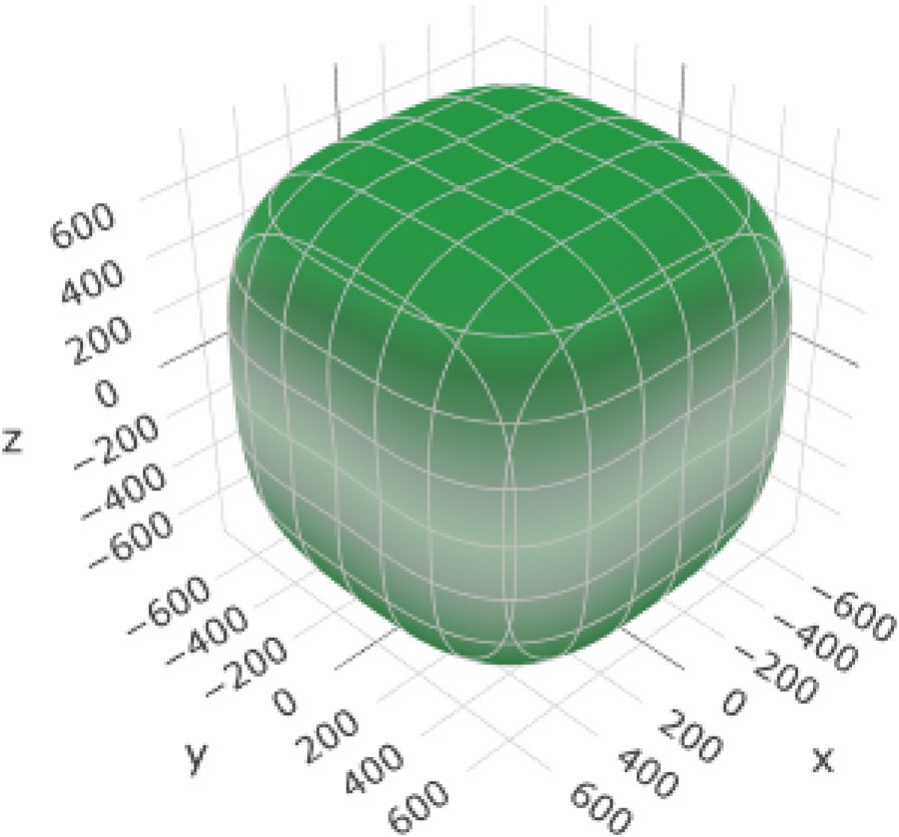
3D representation of Young’s modulus of Yttrium-Stabilized Zirconia

Young’s modulus (*E*) represents the stiffness of the material (Table 8, Fig. 11, and (Figure S5 for the 2D representation). The variation from E_min_ (716.92 GPa) to *E*_max_ (932.53 GPa) allows for controlled stiffness in different directions. This is crucial for mimicking the mechanical behavior of natural teeth and ensuring that the esthetic crown exhibits an appropriate level of rigidity.

**Table 8:** Variations in elastic moduli of yttrium-stabilized zirconia

	Young's modulus		Linear compressibility		Shear modulus		Poisson’s ratio		
	*E*_min_	*E*_max_	*β*_min_	*β*_max_	*G*_min_	*G*_max_	*ν*_min_	*ν*_max_	
Value	716.92 GPa	932.53 GPa	0.92437 TPa^–1^	0.92437 TPa^–1^	306.73 GPa	436.17 GPa	− 0.0057521	0.20403	Value
Anisotropy	1.301		1		1.422		∞		Anisotropy
Axis	0	0.5774	0.7934	0.483	0.7071	0	0.7071	− 0.7071	Axis
	0	0.5774	0	0.6294	0.0001	0	− 0.0002	0	
	1	0.57	0.608	− 0.60	− 0.7071	1	0.7071	0.7071	
					− 0.7071	0.766	0.7071	0	Second axis
					− 0.0002	0.642	− 0.0006	− 1	
					− 0.7071	0	− 0.7071	0

**Fig. 12:**
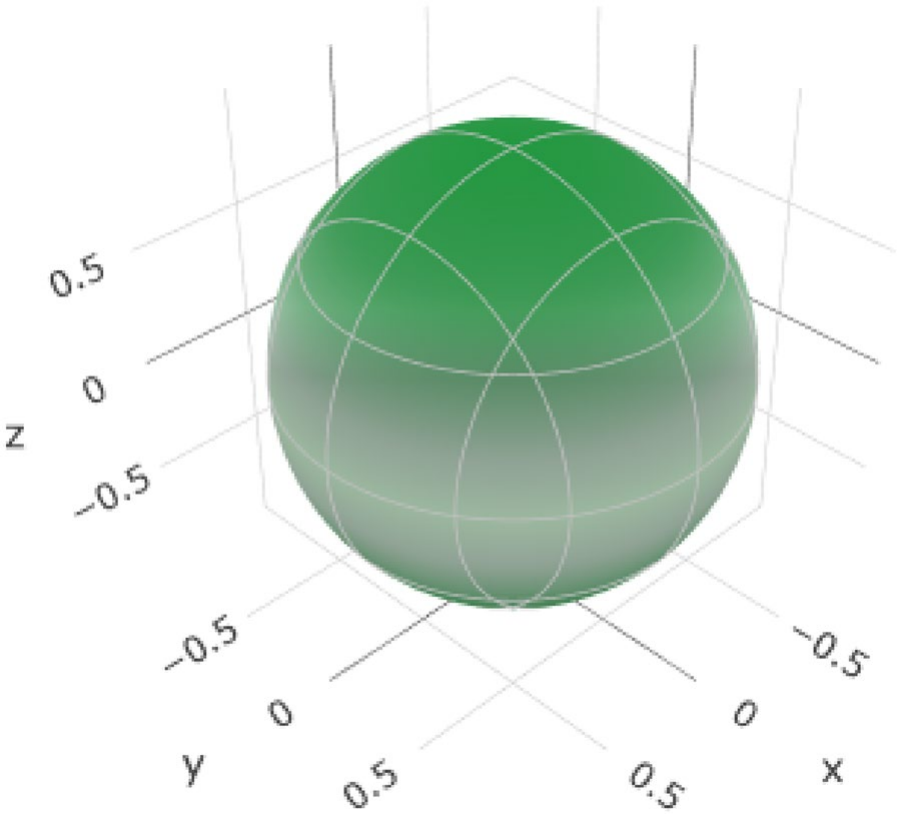
3D representation of the linear compressibility of Yttrium-Stabilized Zirconia

The constant values of linear compressibility (*β*_min_ and *β*_max_ at 0.92437 TPa^–1) indicate a consistent response to compressive stress (Fig. 12 and (Additional file 1: Figure S6 for 2D representation). In esthetic crown applications, where the material may experience compressive forces during biting and chewing, predictable linear compressibility is essential for stability and reliability.

**Fig. 13:**
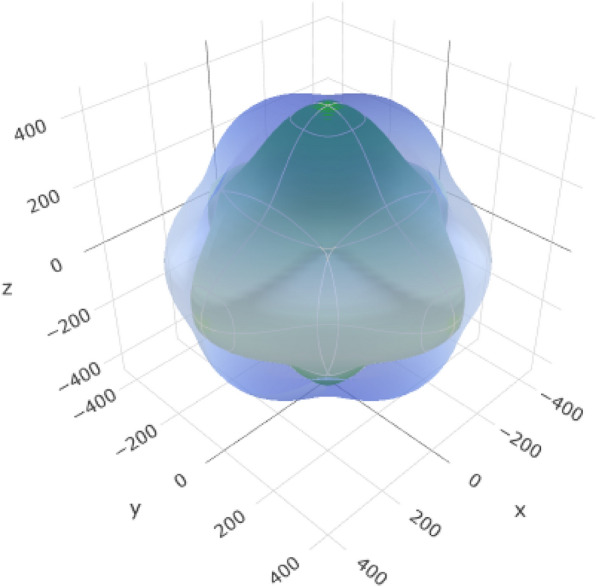
3D representation of the shear modulus of Yttrium-Stabilized Zirconia

The variation in shear modulus (*G*_min_ to *G*_max_ from 306.73 GPa to 436.17 GPa) reflects YSZ's ability to resist deformation under shear stress (Fig. 13 and (Additional file 1: Figure S7 for 2D representation). This property is critical for ensuring that the esthetic crown maintains its structural integrity, especially in areas where shear forces are applied during mastication.

**Fig. 14:**
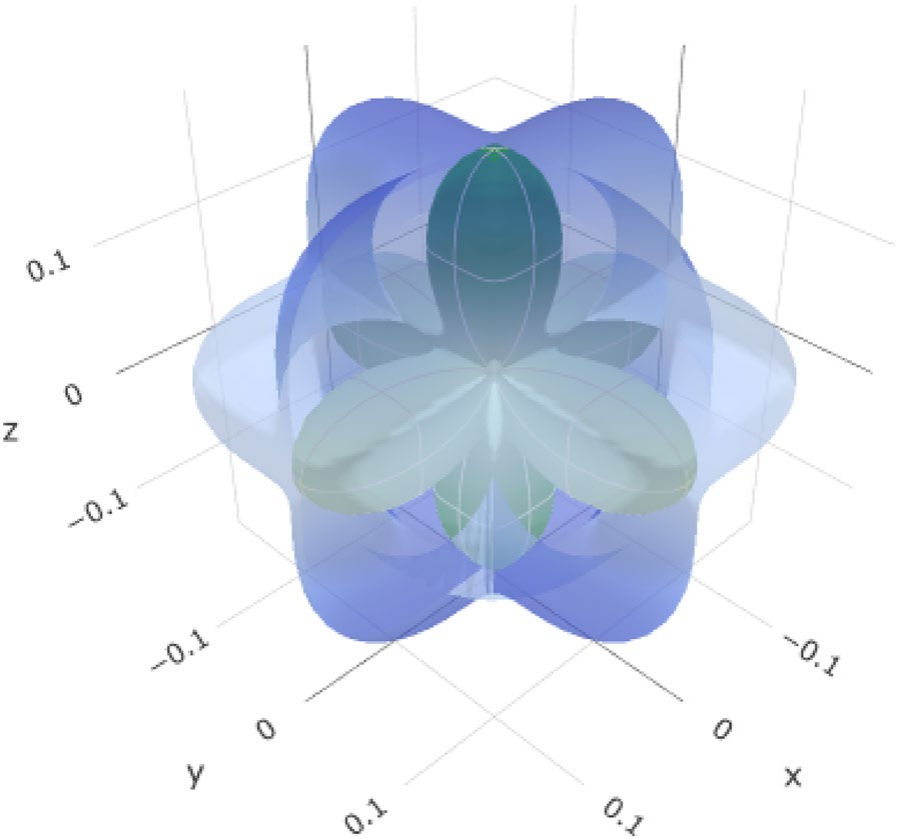
3D representation of Poisson’s ratio of Yttrium-Stabilized Zirconia

The range of Poisson's ratio values (*ν*_min_ to *ν*_max_ from − 0.0057521 to 0.20403) provides insights into the response of YSZ to longitudinal compression (Fig. 14 and (Additional file 1: Figure S8 for 2D representation). Understanding the lateral contraction behavior is vital for preventing dimensional changes and maintaining the stability of the esthetic crown. The anisotropy values and axis information help align the crown orientation with the optimal mechanical properties of YSZ in specific directions. This enables manufacturers to customize crown structures based on the anisotropic nature of materials.

Overall, these variations in elastic moduli allow tailoring of the mechanical properties of YSZ to meet the specific demands of esthetic crown applications. This material can be designed to provide the right balance between stiffness, compressibility, shear resistance, and dimensional stability, thereby ensuring the long-term success and functionality of dental restorations. YSZ generally exhibits higher Young’s modulus, shear modulus, and anisotropy values than alumina.

## Conclusion

Overall, YSZ and alumina possess different strengths and advantages when used in esthetic crowns, and the former seems to be a promising material with high mechanical strength, stable optical properties, and geometries. Alumina, on the other hand, shows a unique CKP geometry, as well as stable band structures and esthetically desirable optical properties, making it suitable for use in esthetic crown designs. In conclusion, dental practitioners should have knowledge of the scientific basis for material selection; however, the best choice may ultimately be specific to individual cases, requiring a delicate balancing act.

### Supplementary Information

**Additional file1 : Fig. S1. ** 2D representation of Young's modulus of Alumina in xy, xz and yz plane. **Fig. S2. ** 2D representation of linear compressibility of Alumina in xy, xz and yz plane. **Fig. S3.** 2D representation of Shear modulus of Alumina in xy, xz and yz plane. **Fig. S4. ** 2D representation of Poisson's ratio of Alumina in xy, xz and yz plane. **Fig. S5. ** 2D representation of Youngs’s modulus of Yttrium-Stabilized Zirconia in xy, xz and yz plane. **Fig. S6. ** 2D representation of linear compressibility of Yttrium-Stabilized Zirconia in xy, xz and yz plane. **Fig. S7. ** 2D representation of Shear modulus of Yttrium-Stabilized Zirconia in xy, xz and yz plane. **Fig. S8. ** 2D representation of Poisson’s ratio of Yttrium-Stabilized Zirconia in xy, xz and yz plane.

## Data Availability

The data are available upon genuine request.

## References

[1] Coffman C, Visser C, Soukup J (2019). mjwsvdp practice crowns and prosthodontics.

[2] Malament KA, Goldstein RE, Stappert CF, Taheri M, Sing. Crown Restorations. In: Ronald E Goldstein’s Esthetics in Dentistry. Hoboken, NJ, USA: John Wiley & Sons, Inc. 2018;498–540.

[3] Ganvir KD, Khangar VJ. Technology: study on fatigue strength of a dental crown: a review. Int J Eng ResTechnol. 2014.

[4] Oueis R (2022). Clinical decision-making: a survey on the influence of specialty and experience in treatment planning of multidisciplinary cases.

[5] Saini RS, Gurumurthy V, Quadri SA, Bavabeedu SS, Abdelaziz KM, Okshah A, Alshadidi AAF, Yessayan L, Mosaddad SA, Heboyan A (2024). The flexural strength of 3D-printed provisional restorations fabricated with different resins: a systematic review and meta-analysis. BMC Oral Health.

[6] Kasem AT, Tribst JPM, Abo-Madina M, Al-Zordk W (2023). Evaluation of different designs for posterior cantilever zirconia inlay-retained fixed dental prostheses in missing tooth replacement: stage one results with 18-month follow-up assessment. J Dentistry.

[7] Madfa AA, Almansour MI, Alshammari AF, Alenezi NM, Alrashidi EF, Aldhaban AA, Aljohani T, Alshammari FA, Alshammari A, Alshammari FA (2023). Knowledge and awareness of dental practitioners about the utilization of endocrown in post-endodontic management. Cureus.

[8] Alshadidi AAF, Alshahrani AA, Aldosari LIN, Chaturvedi S, Saini RS, Hassan SAB, Cicciù M, Minervini G (2023). Investigation on the application of artificial intelligence in prosthodontics. Appl Sci.

[9] Augusti D, Augusti G (2014). Prosthetic restoration in the single-tooth gap: patient preferences and analysis of the WTP index. Clin Oral Implants Res.

[10] Chen Y-W, Moussi J, Drury JL, Wataha JC (2016). Zirconia in biomedical applications. Expert Rev Med Dev.

[11] Saeed F, Muhammad N, Khan AS, Sharif F, Rahim A, Ahmad P, Irfan MJMSCE (2020). Prosthodontics dental materials: from conventional to unconventional. Mater Sci Eng.

[12] Wataha JC (2002). Alloys for prosthodontic restorations. J Prosthetic Dentistry.

[13] Kaur K, Suneja B, Jodhka S, Saini RS, Chaturvedi S, Bavabeedu SS, Alhamoudi FH, Cicciu M, Minervini G (2023). Comparison between restorative materials for pulpotomised deciduous molars: a randomized clinical study. Children.

[14] Felgner S, Henschke C (2023). Patients’ preferences in dental care: a discrete-choice experiment and an analysis of willingness-to-pay. PLOS ONE.

[15] Nayana P, Dhakshaini M, Raghavendra SK, Sowmya S, Ravi M (2019). An evaluation of factors affecting patient's decision making regarding dental prosthetic treatment. Isra Med J.

[16] Kalsi JS, Hemmings KW (2013). The influence of patients decisions on treatment planning in restorative dentistry. Dental Update.

[17] Xia X (2010). Computational modelling study of yttria-stabilized zirconia.

[18] Wei C-C: Yttria stabilised zirconia (YSZ) membranes and their applications. 2009.

[19] Chaopradith DT, Scanlon DO, Catlow CRA (2015). Adsorption of water on yttria-stabilized zirconia. J Phys Chem.

[20] Pal N, Dutta G, Kharashi K, Murray E (2022). Investigation of an impedimetric LaSrMnO3-Au/Y2O3-ZrO2-Al2O3 composite NOx sensor. Materials.

[21] Conrad HJ, Seong W-J, Pesun I (2007). Current ceramic materials and systems with clinical recommendations: a systematic review. J Prosthetic Dentistr.

[22] Babu PJ, Alla RK, AlluriDatla VR, Konakanchi SR (2015). Dental ceramics: part I-an overview of composition, structure and properties. Am J Mater Eng Technol..

[23] Bajraktarova-Valjakova E, Korunoska-Stevkovska V, Kapusevska B, Gigovski N, Bajraktarova-Misevska C, Grozdanov A (2018). Contemporary dental ceramic materials, a review: chemical composition, physical and mechanical properties, indications for use. Open access Macedonian J Med Sci.

[24] Francis NT (2019). To evaluate the effect of adding different grain size aluminium oxide particles on flexural strength and surface finish of pmma denture base resin: an in vitro study.

[25] Czepułkowska W, Wołowiec-Korecka E, Klimek L (2018). The role of mechanical, chemical and physical bonds in metal–ceramic bond strength. Archiv Mater Sci Eng.

[26] Helvey A (2014). Classifying dental ceramics: numerous materials and formulations available for indirect restorations. Compendium.

[27] Shi HY, Pang R, Yang J, Fan D, Cai H, Jiang HB, Han J, Lee E-S, Sun Y (2022). Overview of several typical ceramic materials for restorative dentistry. BioMed Res Int.

[28] Burger W, Kiefer G (2021). Alumina, zirconia and their composite ceramics with properties tailored for medical applications. J Compos Sci.

[29] Giordano R (2022). Ceramics overview. Br Dental J.

[30] Rehman J, Muhammad Usman M, Tahir B, Abid Hussain M, Rehman A, Ahmad N, Alrobei H, Shahzad K, Ali AM, Muhammad S (2021). First-principles calculations to investigate structural, electronic and optical properties of Na based fluoroperovskites NaXF3 (X= Sr, Zn). Solid State Commun.

[31] Yaakob M, Taib M, Hassan O, Yahya M (2015). Low-energy phases, electronic and optical properties of Bi1−xLaxFeO3 solid solution: Ab-initio LDA+ U studies. Ceram Int.

[32] Köcher SS, Schleker P, Graf M, Eichel R-A, Reuter K, GranwehrScheurer J (2018). Chemical shift reference scale for Li solid state NMR derived by first-principles DFT calculations. J Magn Resonan.

[33] Saini RS, Mosaddad SA, Heboyan A (2023). Application of density functional theory for evaluating the mechanical properties and structural stability of dental implant materials. BMC Oral Health.

[34] Saputro DRS, Widyaningsih P (2017). Limited memory Broyden–Fletcher–Goldfarb–Shanno (L-BFGS) method for the parameter estimation on geographically weighted ordinal logistic regression model (GWOLR).

[35] Wan P, Zhao N, Qi F, Zhang B, Xiong H, Yuan H, Liao B, Ouyang X (2020). Synthesis of PDA-BN@ f-Al2O3 hybrid for nanocomposite epoxy coating with superior corrosion protective properties. Progress Organ Coatings.

[36] Hrubý V, Zdražil L, Dzíbelová J, Šedajová V, Bakandritsos A, Lazar P, Otyepka M (2022). Unveiling the true band gap of fluorographene and its origins by teaming theory and experiment. Appl Surf Sci.

[37] Yoshikawa K, Kataoka Y, Kobayashi M, Yamaguchi M, Ogawa H, Miyazaki T (2018). MANABE refractive index measurement of dental materials by swept-source optical coherence tomography. Jpn J Conserv Dent.

[38] Hertzberg RW, Vinci RP, Hertzberg JL (2020). Deformation and fracture mechanics of engineering materials.

