# Supplementary material for: Quantum mechanical analysis of yttrium-stabilized zirconia and alumina: implications for mechanical performance of esthetic crowns
Source: Eur J Med Res. 2024 Apr 24;29:254. doi: 10.1186/s40001-024-01851-2 (PMC11044456; doi:10.1186/s40001-024-01851-2)
Supplement: Supplementary file 1 — Additional file1 : Fig. S1. 2D representation of Young's modulus of Alumina in xy, xz and yz plane. Fig. S2. 2D representation of linear compressibility of Alumina in xy, xz and yz plane. Fig. S3. 2D representation of Shear modulus of Alumina in xy, xz and yz plane. Fig. S4. 2D representation of Poisson's ratio of Alumina in xy, xz and yz plane. Fig. S5. 2D representation of Youngs’s modulus of Yttrium-Stabilized Zirconia in xy, xz and yz plane. Fig. S6. 2D representation of linear compressibility of Yttrium-Stabilized Zirconia in xy, xz and yz plane. Fig. S7. 2D representation of Shear modulus of Yttrium-Stabilized Zirconia in xy, xz and yz plane. Fig. S8. 2D representation of Poisson’s ratio of Yttrium-Stabilized Zirconia in xy, xz and yz plane. [file 40001_2024_1851_MOESM1_ESM.docx]

**Additional file Informations**

**Quantum Mechanical Analysis of Yttrium-Stabilized Zirconia and Alumina: Implications for Esthetic Crown Mechanical Performance.**


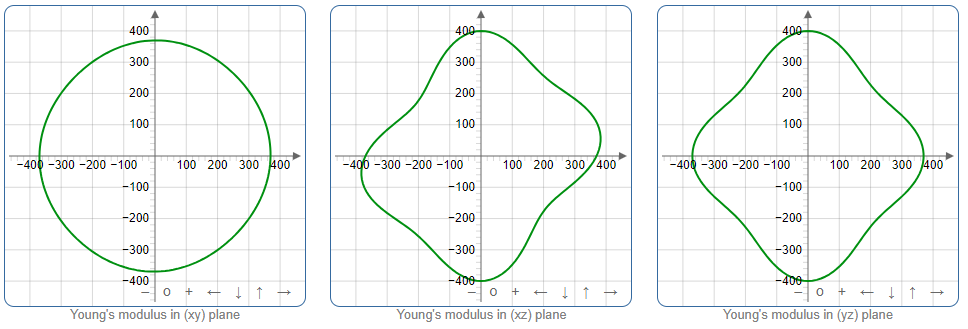


Figure S1: 2D representation of Young's modulus of Alumina in xy, xz and yz plane.


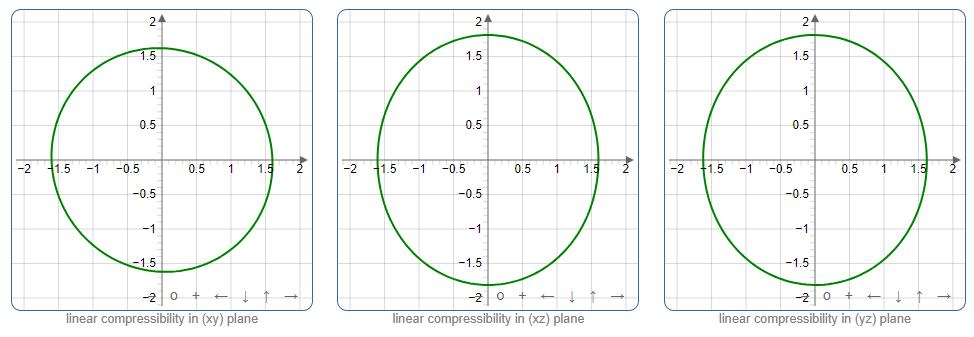


Figure S2: 2D representation of linear compressibility of Alumina in xy, xz and yz plane.


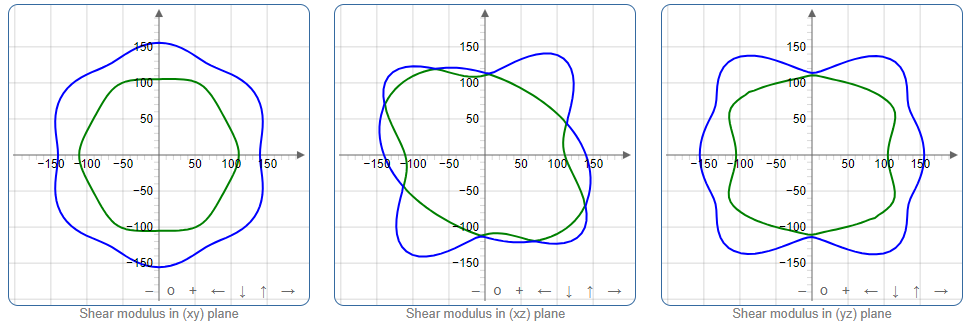


Figure S3: 2D representation of Shear modulus of Alumina in xy, xz and yz plane.


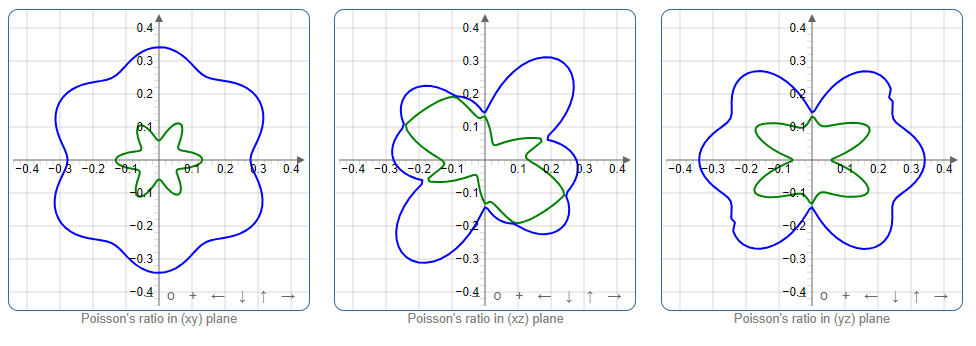


Figure S4: 2D representation of Poisson's ratio of Alumina in xy, xz and yz plane.


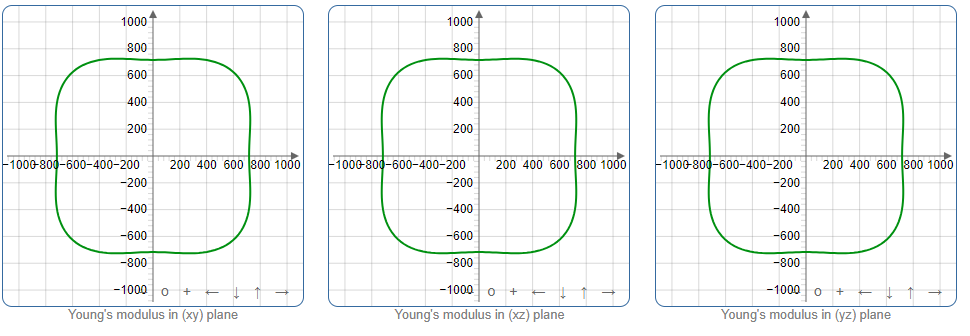


Figure S5: 2D representation of Youngs’s modulus of Yttrium-Stabilized Zirconia in xy, xz and yz plane.


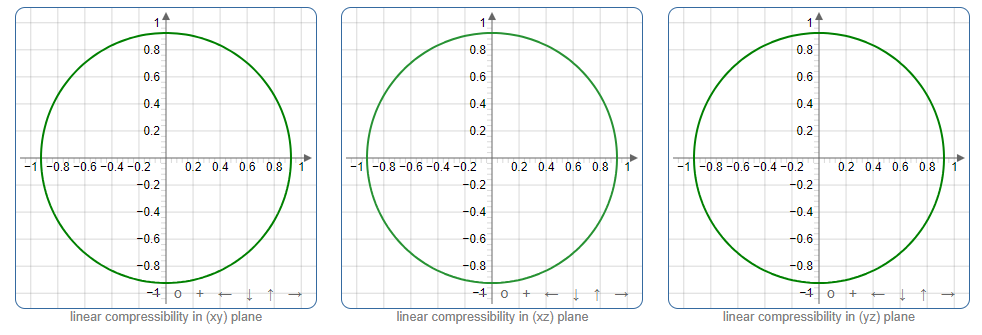


Figure S6: 2D representation of linear compressibility of Yttrium-Stabilized Zirconia in xy, xz and yz plane.


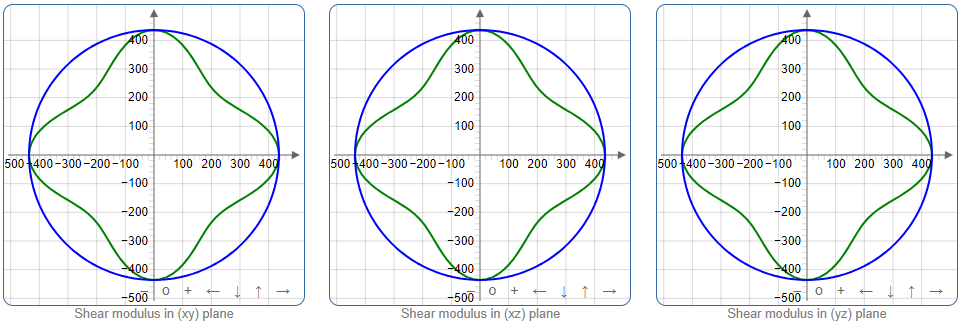


Figure S7: 2D representation of Shear modulus of Yttrium-Stabilized Zirconia in xy, xz and yz plane.


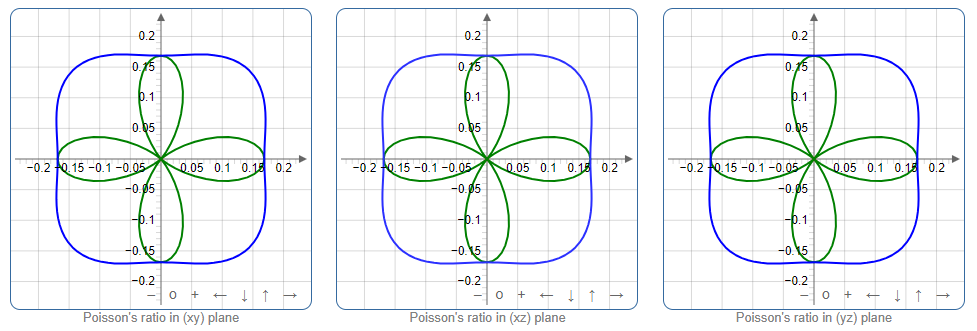


Figure S8: 2D representation of Poisson’s ratio of Yttrium-Stabilized Zirconia in xy, xz and yz plane.
